# The Satiating Effect of Extruded Plant Protein Compared with Native Plant and Meat Protein in a Ragú “Bolognaise” Meal: A Randomized Cross-Over Study

**DOI:** 10.3390/nu16193407

**Published:** 2024-10-08

**Authors:** Mille Skov Martin, Anja Weirsøe Dynesen, Birthe Petersen, Iben Lykke Petersen, Patrícia Duque-Estrada, Margit Dall Aaslyng

**Affiliations:** 1 Department of Nutrition and Health, Centre for Health and Rehabilitation, University College Absalon, 4200 Slagelse, Denmark; mism@pha.dk (M.S.M.); andy@pha.dk (A.W.D.); bpe@pha.dk (B.P.); 2 Department of Food Science, University of Copenhagen, 1958 Copenhagen, Denmark; ilp@food.ku.dk (I.L.P.); patricia.estrada@food.ku.dk (P.D.-E.)

**Keywords:** legume proteins, protein digestibility, satiety, subjective appetite, texturized vegetable protein

## Abstract

Protein increases satiety by, among other things, increasing the content of certain amino acids in the blood. Plant proteins generally have a lower digestibility than meat proteins. The digestibility increases after extrusion; thereby, extrusion potentially also increases the satiating effect. We investigated subjective appetite and ad libitum energy intake (adlib_EI) following ragú “bolognaise” meals with three different protein sources. We hypothesized that the satiating effect of texturized vegetable proteins (TVP) was comparable to that of animal protein (Meat) and that TVPs would have a stronger satiating effect than non-texturized legume proteins (Green). Test meals were theoretically designed to be similar in weight, energy (kJ), macronutrients and fiber. The in vitro protein digestibility (IVPD) and the amino acid composition were analyzed. A randomized, single-blinded, three-way, cross-over study including 25 healthy men was carried out. There were no significant differences between the three meals in terms of subjective appetite. The adlib_EI was significantly lower after the TVP meal (758 kJ) than after the Meat meal (957 kJ), with the Green meal in between (903 kJ). The IVPD was significantly higher in the Meat meal (30.72%) than in the Green meal (20.17%), with the TVP meal in between (21.05%). In conclusion, the TVP meal had a higher long-term satiating effect than a similar meal with meat.

## 1. Introduction

Global meat consumption has increased considerably over the past few decades [1], with meat production leaving a significantly larger environmental and climate footprint than the production of plant-based foods [2]. Furthermore, it is well established that eating a diet high in plants is linked to health benefits, such as a lower risk of type 2 diabetes [3], cardiovascular disease [4] and cancer [5]. Therefore, it is broadly accepted that, due to the environmental impact and population health consequences, it is necessary to shift from animal-based diets towards more plant-based diets [6,7]. Shifting to a more plant-based diet is associated with certain challenges. For example, consumers feel that “not getting enough protein” and “not becoming full” are barriers to eating plant-based foods [8,9]. 

Legumes have a high protein content which is superior to that of most other plant foods. Therefore, legumes have become a significant source of protein in the transition to a more plant-based diet. Legumes include crops like beans (including soy beans) and peas, chickpeas and faba beans, all having a high protein content. However, proteins from plants generally have a lower nutritional quality than animal proteins. Meat and dairy products are excellent sources of high nutritional quality proteins, meaning that animal proteins are easily digestible and can provide sufficient levels of indispensable amino acids (IAAs) [10]. Individual plant proteins, however, usually fail to provide sufficient levels of IAAs and have a lower digestibility due to anti-nutritional factors such as protease inhibitors [11]. 

Well-established ways of improving the nutritional quality of plant proteins include the use of (1) blends of plant protein sources to ensure a balanced IAA profile and (2) food processing to inactivate anti-nutritional factors and unfold the native structure of the proteins, thereby improving digestibility [12,13,14,15]. One such food-processing method is extrusion. Low-moisture extrusion is used to produce texturized vegetable proteins (TVPs) by applying high temperature and mechanical forces. By combining the two approaches, a protein source with a higher protein quality compared with the individual native protein sources can be produced. 

Protein in meals is linked to satiation and satiety in that, at sufficiently high levels, protein is superior to equivalent quantities of energy from carbohydrate and fat in terms of stimulating these sensations [16,17]. Satiation refers to the process which leads to the completion of a meal, while satiety refers to the feeling of fullness after a meal and in the time that follows [18]. The satiating effect covers both satiation and satiety. 

Protein intake induces complex signaling in the gut and following absorption, which affects the sensation of satiety [17,19]. Thus, products derived from protein digestion are superior to other nutrients in terms of releasing gastrointestinal hormones that are relevant to satiety [19]. Furthermore, an increase in the concentrations of amino acids in the circulating blood is another mechanism described for protein-induced satiety [20], with specific amino acids, such as ketogenic and branched-chain amino acids, being linked to increased satiety by increasing the ketone body concentration in the blood [17,21]. Although it has been described that both the quality and type of protein seem to be involved in the sensation of satiety, the literature cannot present clear evidence that indicates whether there is a difference in the satiating effect of animal versus plant protein [17]. In order to overcome consumer beliefs including concerns about the protein content and the satiating effect of plant-based meals, these types of meals must deliver high-quality protein and optimal satiety.

In the present study, we hypothesized that a meal based on TVPs with reduced trypsin inhibitor activity and therefore increased protein digestibility would have a satiating effect comparable to a meal based on easily digestible animal protein. Furthermore, we hypothesized that, due to the increased protein digestibility of the TVPs, the meal based on TVPs would have a stronger satiating effect than a meal based on non-texturized legume protein. 

The aim of the present study was to investigate subjective appetite and ad libitum energy intake following a meal based on TVPs with a complete IAA profile based on a mixture of peas and oats, and to compare it to (1) a meal based on animal protein (beef) and (2) a meal based on legumes, in this case soybeans.

## 2. Materials and Methods

### 2.1. Study Design

This study was designed as a randomized, single-blinded, three-way cross-over study and was carried out at the Department of Nutrition and Health, at University College Absalon, Slagelse, Denmark, in April–June 2023. Participants were randomly assigned to a sequence of three test meals using a randomized block design. Each test meal was separated by a period of at least five days. The participants were instructed to maintain their normal diet and lifestyle between the test days. They were also instructed to abstain from vigorous physical activity and alcohol consumption for 24 h preceding each test day. 

This study was conducted in accordance with the Declaration of Helsinki [22], and the Institutional Ethics Committee of University College Absalon approved the protocol (Ref. No. 115229). Furthermore, the Regional Ethical Committee of Zealand confirmed that no formal approval by the committee was required (EMN-2023-00848).

### 2.2. Measurements

Subjective appetite was assessed using computerized 100 mm visual analogue scales (VAS). Data were collected via electronic tablets using RedJade Sensory Software (Martinez, CA, USA, 2023). Subjective appetite assessments included satiety, hunger, fullness and prospective food intake. Each VAS was anchored with the most positive and the most negative rating for each participant’s sensation. A translation into Danish was used for the following four questions describing each sensation: (1) Hunger: “How hungry are you?”, anchored with “Not hungry at all” and “As hungry as I have ever felt”; (2) Satiety: “How satisfied do you feel?”, anchored with “Completely empty” and “Cannot eat another bite”; (3) Fullness: “How full do you feel?”, anchored with “Not full at all” and “Totally full”; (4) Prospective food intake: “How much do you think you can eat?”, anchored with “Nothing at all” and “A large amount”. A description can be seen in the Supplementary Materials, Table S1.

An ad libitum meal was served 150 min after the test meal. The amount consumed was weighed and converted into kJ.

### 2.3. Participants 

Participants were recruited in three ways: through posters and flyers at University College Absalon, Campus Slagelse, and the University of Southern Denmark, Campus Slagelse, by social media (Facebook, Instagram and LinkedIn) and by word of mouth. 

The inclusion criteria were healthy men, normal to moderate overweight (body mass index (BMI) 22–29.9 kg/m^2^), 18–64 years of age. The exclusion criteria were daily intake of prescription medicine known to affect appetite, dislike of specific foods, a special diet restricting intake of the study meals, food allergy, vigorous physical activity (≥10 h/week), dieting and an impaired ability to feel satiety. 

Height and weight were measured in order to calculate the BMI. Height was measured on a Seca 217 to the nearest 0.5 cm (participants were asked to take off their shoes). Weight was measured on a Tanita DC360 to the nearest 0.1 kg (participants were asked to take off their shoes and heavy clothes, e.g., jumper or jacket). Questions related to their activity level as well as other inclusion and exclusion criteria were self-reported.

A total of 29 subjects were screened; two did not meet the inclusion/exclusion criteria, and two withdrew their consent before starting or during the study. Thus, 25 participants were enrolled in this study. The characteristics of the participants are shown in Table 1. The participants were given verbal and written information, and informed consent was obtained. Participants received approximately EUR 135 as compensation on completion of all three test days. 

The sample size for this study was based on power calculations conducted by Flint et al. [23]. It was estimated that, in order to detect a 10% difference in satiety measurements using VAS with a statistical power of 90% and a significance level of 0.05, a population of minimum *n* = 24 was required.

### 2.4. Meals

#### 2.4.1. Standardized Evening Meal 

The standardized evening meal consisted of lasagna (beef, Løgismose), a wholegrain wheat bread roll (Primitivo, Il Fornaio) and butter (Lurpak). The meal was individualized according to the estimated energy intake for each participant and was equivalent to 30% of their recommended daily intake [24]. All participants received one lasagna, while the energy content of the meal was adjusted with the quantity of bread rolls and butter.

#### 2.4.2. Standardized Breakfast 

The standardized breakfast consisted of a wholegrain wheat bread roll (Primitivo, Il Fornaio), butter (Lurpak), sliced chicken ham (Tulip), raspberry jam (Skælskør Frugtplantage), yoghurt (pear and banana, Arla) and apple juice (Premium, Rynkeby) with a total energy intake of approximately 2 MJ. The participants were also given the option of coffee, tea or water (0.15 L) without milk or sugar. The option was noted and repeated on all three test days for the same participant.

#### 2.4.3. Test Meals Composition

The three test meals consisted of pasta (97 g boiled pasta made from 50 g dry pasta) and “bolognaise” containing different main sources of proteins: (1) texturized pea protein and oat protein (TVP) (567 g); (2) beef (Meat) (562 g); and (3) soybeans (Green) (562 g) (Table 2). The meals were designed to be similar in weight, energy content and composition of macronutrients, including dietary fiber. 

The meals were designed using the dietary assessment tool VITAKOST™ (MADLOG ApS, Kolding, Denmark). 

#### 2.4.4. Ad Libitum Meal Composition

The ad libitum meal consisted of quiche with potatoes and carrots, with an energy content of 434 kJ/100 g. A whole quiche (approximately 675 g) was served to each participant and was weighed both before and after consumption.

### 2.5. Procedure

#### 2.5.1. The Days Prior to the Test Days

The evening before each test day, the participants were provided with a standardized evening meal. The meal was consumed at 7.00 p.m. at home (Table 3) with no restrictions on water intake. After consumption of the meal, the participants fasted. Water intake was restricted to 0.5 L during fasting.

#### 2.5.2. Test Days

A standardized breakfast was served to the participants at 8.00 a.m. at the test meal facility on each test day. The breakfast was consumed in 15 min. Afterwards, the participants were free to work, attend lectures on campus, etc. in the hours before the test. A bottle of water (0.33 L) was provided for consumption during this period, and the participants were instructed to fast.

The participants returned to the test meal facility at 11.45 a.m., and a brief introduction was given. The participants filled in a VAS at time points 0, 15, 30, 60, 90, 120 and 150 min (t = 0–150). Assessment t = 0 was given at 12.00 p.m., and then, the test meal and a glass of water (0.23 L) were served. The meal had to be consumed before the second assessment was performed (t = 15).

At t = 60, another glass of water (0.23 L) was served and had to be consumed within 30 min. An ad libitum meal was served with an optional glass of water (0.23 L) after the final VAS assessment (t = 150), and the participants were asked to eat at a steady pace until they felt comfortably satiated.

### 2.6. In Vitro Protein Digestibility (IVPD)

This study assessed gastrointestinal protein digestion using a static, multistep IVPD assay based on the method described by Joehnke et al. (2018) [25]. Before the assay, samples were freeze-dried and normalized to contain 50 mg of protein and solubilized in 10 mL of 0.05 M HCl overnight at 5 °C. Internal standards, sample reference and sample blank were prepared in the same manner as the samples with bovine serum albumin (BSA, as a reference) and free alanine (used as an internal standard), and for the blank samples, only 0.05 M HCl was added. The following day, the gastric phase was simulated by adding a freshly prepared 1 mg/mL pepsin solution (in 0.05 M acetate buffer, pH 4.5, 920 U/mg protein from porcine gastric mucosa) to the samples with an enzyme-to-substrate ratio of 1:50 *w*/*w* and incubation for one hour at 37 °C and 80 rpm in a shaking water bath. The pH was maintained below 2.0. Subsequently, the small intestinal phase was simulated by adding 0.6 M sodium bicarbonate buffer, 1.15 M NaOH and 100 mg/mL sodium cholate hydrate solution to the residual digesta. Additionally, a freshly prepared 2 mg/mL pancreatin solution (porcine pancreatin in 1 mM HCl) was added with an enzyme-to-substrate ratio of 1:10 *w*/*w*, and the samples were incubated for an additional two hours under the same conditions as before. Aliquots were withdrawn before and after each digestion stage, and protein hydrolysis was stopped by adding sodium borate buffer (0.05 M, pH 10.0) to the samples, and stored at 5 °C for further analysis. The %IVPD was evaluated by measuring the free α-amino groups released during each digestion stage utilizing the trinitrobenzenesulfonic acid (TNBS) method in a 96-well plate and microplate reader (Epoch 2, Biotek Instruments, Inc., Winooski, VT, USA). Samples were diluted in borate buffer (0.05 M, pH 10), after which TNBS (0.1% 2,4,6-TNBS in water) was added to samples, calibration curve and blank. The reaction between TNBS and primary α-amino groups was monitored continuously at 37 °C for ten minutes at 450 nm. Absorbance data were analyzed using Gen5 v.3.11 Data Analysis Software (BioTek Instruments, VT). The concentration of α-amino groups was determined from a standard curve prepared with a stock solution of 0.2 mg/mL alanine (0.05 M borate buffer, pH 10) and corrected using a plate blank containing only borate buffer. The %IVPD was calculated in relation to the alanine internal standard with corrections for enzyme-only blanks. Each sample underwent digestion in triplicate. 

### 2.7. Chemical Composition and Amino Acid Analysis

The macronutrient composition (protein, fat, carbohydrate, dietary fiber, water, ash and energy) was analyzed in duplicate for two different meals per meal type by an accredited laboratory (Société Générale de Surveillance SA (SGS Analytics), Malmö, Sweden) after the test days.

The amino acid composition was analyzed by the same laboratory for one meal per meal type.

### 2.8. Statistical Analysis

All calculations were performed using R and RStudio (2022.02.2 Build 485). The analyzed content of macronutrients and energy of the three test meals was compared using a one-way analysis of variance with meal type as a fixed effect.

Initially, the data quality of the VAS measurements was checked. If one assessment was clearly an outlier, indicating that the scale seemed to have been reversed by the participant for that exact measurement, a new value was calculated as the mean of the time point before and the time point after. This was performed for 11 out of a total of 700 assessments.

For the four subjective appetite assessments measured using VAS, the difference between the measurement before and after the test meal (T0 and T15) was analyzed using a one-way analysis of variance (ANOVA) with meal type as a fixed effect and participant as a random effect.

The area under the curve (AUC) was calculated for satiety and prospective eating and the area over the curve (AOC) for hunger and fullness for the interval T15 until T150 using the trapezoidal method (DescTools package). Initially, the correlation between the AUC/AOC and the BMI was calculated for all three test meals together. The correlation was 0.2 for all four AUC/AOCs. The difference between the three test meals in the AUC/AOC was then analyzed by a mixed-model ANOVA with meal type, test day and their interaction as fixed effects and participant as a random effect. Since there were no correlations between the BMI and the AUC/AOC in the three test meals, the BMI was not included in the model. Furthermore, since the interaction was insignificant (*p* = 0.26), the model was reduced to include only the main effects. 

The repeated measurement was analyzed using a mixed-model ANOVA (Imer package), with test meal, time and the interaction as fixed effects adjusting for baseline (T0) and participant as a random effect. The optimal correlation structure (linear, exponential or Gaussian) was the exponential for satiety, hunger and fullness and the linear for prospective food intake, which was taken into account in the model. As the interaction was insignificant, the model was reduced to include only the main effects.

Ad libitum intake was analyzed by a mixed-model ANOVA, with meal type and test day and their interaction and BMI as fixed effects and participant as a random effect. The model was stepwise reduced, initially removing the BMI and then the interaction, giving a final model with meal type and test day as fixed effects and participant as a random effect.

For all analyses, a pairwise comparison was calculated for significant effects using Tukey–Kramer post hoc comparison with multiplicity adjustment (Package emmeans).

The IVPD results are presented as the mean and standard deviation. A one-way analysis of variance (ANOVA), followed by Tukey’s post hoc test, was performed using GraphPad Prism (GraphPad Software v. 10.1.0). Statistical significance was considered at *p* < 0.05.

## 3. Results

To investigate the effect of texturized plant protein compared with legume protein and meat protein on subjective appetite, three meals with theoretically comparable nutritional composition were designed. The meals were compared with regard to the analyzed nutritional composition and effect on subjective appetite assessed by VAS and by energy intake in an ad libitum meal. 

### 3.1. Nutritional Composition of the Meals

The meals were designed theoretically to have a similar content of protein, dietary fiber, energy and weight. This was achieved for protein, energy and weight (Table 4). However, the content of dietary fiber was significantly lower in the Meat meal compared with the Green meal (*p* = 0.02). There were no significant differences in the dietary fiber content between the TVP meal and the other meals (*p* (Green-TVP) = 0.082, *p* (Meat-TVP) = 0.138). 

To further characterize the meals, the in vitro protein digestibility (IVPD) and the amino acid composition were analyzed (Table 5). The IVPD was higher in the Meat meal than in the Green meal (*p* = 0.0247), though not different from the TVP meal (*p* = 0.0832). No difference was seen between the TVP and the Green meal. In contrast, the Meat bolognaise (without pasta) differed significantly from both the TVP and the Green bolognaise (*p* < 0.001) and with no significant difference between the TVP and the Green bolognaise (*p* = 0.4289).

Comparing the IVPD of the meals (ragú “bolognaise”) with the “bolognaise” itself (without the pasta) showed that, for the TVP meal, the digestibility was lower in the meal compared with the bolognaise itself, while the IVPD was slightly increased in the Meat meals, although the differences were insignificant. Further details on the IVPD are given in the Supplementary Materials, Table S2.

All meals had an adequate amino acid composition in line with the WHO requirement for indispensable amino acids [26]. However, there were some differences between the meals: (1) the TVP meal was low in the sulfur-containing amino acids cysteine (Cys) and methionine (Met) but high in threonine (Thr), (2) the Meat meal was high in histidine (His) but low in phenylalanine (Phe) and tyrosine (Tyr), (3) the Green meal was low in lysine (Lys) content in particular. Furthermore, hydroxyproline (HPro) was only present in the Meat meal. The sum of ketogenic amino acids was also different between the three meals, with the highest levels in the TVP meal followed by the Meat meal and lastly the Green meal.

### 3.2. Subjective Appetite

**Figure 1:**
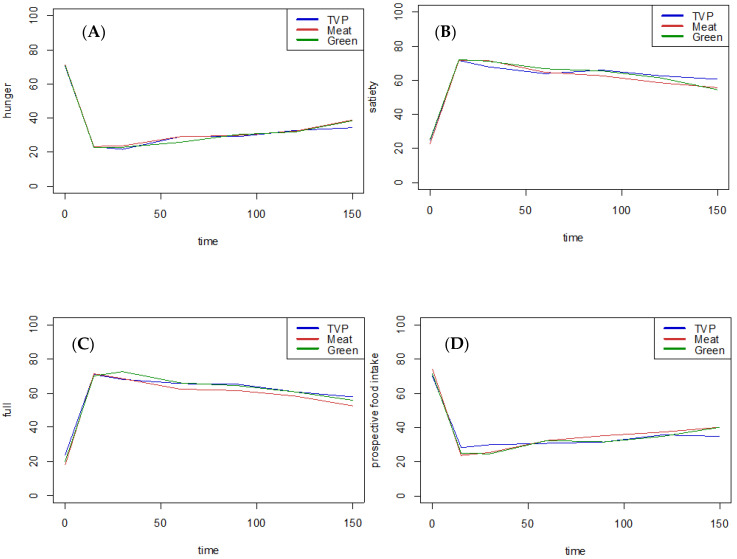
Hunger (**A**), satiety (**B**), full (**C**) and prospective food intake (**D**) measured on a 10 cm VAS scale.

The subjective appetite assessment was carried out before the test meal and up to 150 min after the baseline (Figure 1). The difference between the prior assessment (t = 0) and the assessment immediately after the meal (t = 15) reflects the satiation. No significant differences were seen between the three meals in any of the four attributes (*p* (hunger) = 0.97, *p* (satiety) = 0.88, *p* (fullness) = 0.45, *p* (prospective) = 0.19), showing the same effect on satiation for all meals.

The satiating effects from t = 15 to t = 150 were compared using both the AUC and AOC and by an analysis of variance using repeated measurement (Table 6). Neither of these measurements revealed significant differences between the three meals, indicating that, during this time span, the three meals were equally satiating.

### 3.3. Ad Libitum Meal

The energy intake from the ad libitum meal was measured at t = 150. At this time, a significant difference was seen between the three meals, as the energy intake of the TVP meal was lower than that of the Meat meal (Table 7). The energy intake after the Green meal was higher than after the TVP meal, although not significant (*p* = 0.0975).

## 4. Discussion

### 4.1. Meal Composition

It is well known that the satiating effect of a meal depends on several factors, including the content of energy, protein and dietary fiber, especially beta-glucans [27,28], as well as factors such as meal size, meal variation [29] and hedonic response [30]. To be able to compare the satiating effect of different protein sources, the other factors should therefore be standardized. Nevertheless, many studies have focused on standardizing the energy and protein content [31,32,33,34,35], while only few studies have also standardized the content of dietary fiber [36,37,38].

It was the aim of this study to design three meals that were similar in their content of protein, energy and dietary fiber and also in meal size (g), and where the main protein part came from the meat/TVP or soy beans, in order to investigate the effect of the protein source without any interaction from the other factors. Furthermore, the meals were intended to be as similar in appearance as possible. 

Wheat bran was included in the recipe to the Meat meal to increase the content of dietary fiber. Wheat bran contains a high amount of dietary fiber, mainly insoluble dietary fiber [39]. Despite the addition of wheat bran and similar theoretical calculation of dietary fiber between treatment, there was substantially lower dietary fiber in the Meat meal compared to the Green treatment.

In addition to dietary fiber, wheat bran also contains approximately 18% protein [40] with a high amount of the amino acid Met [41]. This might contribute to the higher concentration of Met in the Meat meal compared with the two vegetable meals. However, since the in vitro protein digestibility of wheat bran is low [42], its incorporation into the Meat meal is not expected to contribute to the digested proteins. Nevertheless, the IVPD was higher compared with the two other “bolognaises”, although lower than BSA, which is considered to be a highly digestible protein (Supplementary Table S2).

The texturized vegetable protein was made from a combination of pea protein (83%) and oat protein (17%). Three factors determined this protein combination. First, the pea protein should be combined with a protein source, which would result in an adequate amino acid combination in the final TVP. Second, the sensory quality should be high, excluding protein sources with a distinctive flavor, and third, the combination should be technologically feasible. 

In the Green meal, we included soybeans as the legume protein source in order to provide sufficient protein without increasing the content of carbohydrates. The digestibility of soy protein is relatively high compared with other plant protein sources such as peas [43]. This might explain the fact that no difference in IVDP was found in this study between the TVP and the Green meal, even though it has been documented that in vitro protein digestibility increases after extrusion [13,15,44].

Soy protein is also known to have a higher content of IAA compared with pea protein [15]. However, the content of specific amino acid Lys is higher in pea than in soy [15], which was reflected in the higher content of Lys in the TVP meal compared with the Green meal. Also, the content of Thr is higher in the TVP meal, although this cannot be readily explained from the composition of the TVP, since neither peas nor oats have a higher content of Thr than soy [45].

One of the hypotheses behind the satiating effect of protein is the content of ketogenic amino acids which can increase the satiating effect [17]. Here, it is of interest that the TVP meal has a higher content of these amino acids compared with the two other meals, followed by the Meat meal, with the lowest content in the Green meal (Table 5).

Apart from these differences, no significant difference was seen between the three test meals in the content of energy, protein, fat and carbohydrate, and, furthermore, the meal size (g) was similar in all three test meals, with a served energy content of 3.1 MJ. 

The energy content of the meal must be chosen with care [30]. A larger meal can induce a higher satiating effect [29,30], thereby masking the difference between meals, since the participants remain full for the test period after all meals. However, a small meal size might mean that the participants are hungry after all meals, and therefore, the meals might not be differentiated. The served energy in this study is in accordance with other meal studies such as [36,46] but lower than [34,37] and higher than [47,48]. Furthermore, not just the energy content but also the size (g) of the meal can have an influence on the satiating effect [29]. In our study, the meal size (g) was larger than, for example, in Kristensen et al. [34] but at the same level as that consumed in another study, in which the participants were asked to eat plant-based and meat-based meals [35].

### 4.2. Meal Experiment

The hypothesis was that the Meat meal and the TVP meal would have a higher satiating effect than the Green meal due to their higher protein quality. However, no differences could be seen in the assessment of satiety, hunger, fullness or prospective eating. Only the ad libitum energy intake was different, being lowest in the TVP meal. This indicates a higher long-term satiating effect of the TVP compared with the two other meals. 

The ad libitum energy intake was significantly lower in the TVP meal than in the Meat meal and, albeit insignificantly, also lower than in the Green meal. Since the IVPD was highest in the meat bolognaise, the in vitro protein digestibility cannot, by itself, explain this difference. The composition of amino acids might explain part of the difference, since the TVP meal had the highest content of ketogenic amino acids, which are hypothesized to participate in the satiety cascade [17]. This stresses the importance of combining different protein sources to achieve an optimal amino acid composition [44]. Another hypothesis could be that the digestion rate is lower in the TVP meal, thereby inducing a higher long-term satiety. Using the analytical method as in this study, only the end-point digestibility is known and not the rate of digestibility, and the hypothesis can therefore not be further explored.

The content of dietary fiber was higher in the two vegetable meals compared with the Meat meal, and, furthermore, the dietary fiber in the Meat meal were primarily from wheat bran, which is high in insoluble fiber and has a lower satiating effect than soluble fiber. This might partly explain the lack of expected difference in satiety between the Meat meal and the Green meal, even though the IVPD was higher in the Meat meal than in the two vegetable meals, since dietary fiber has a satiating effect independent of proteins.

This lack of effect on the subjective appetite score and the lower ad libitum energy intake after a plant-based meal compared with a Meat meal were also seen by [31]. In their study, the content of dietary fiber was 2.4 g/100 g to 4.5 g/100 g, which is close to the 3.75 g/100 g in the Meat meal and 5.55 g/100 g in the Green meal in our study. Another study found no difference between different plant and animal protein sources in either subjective appetite measurement or ad libitum energy intake [37] with a high, standardized dietary fiber content of 28.4–28.8 g/100 g. In contrast, a third study found a difference in both subjective appetite measurements and ad libitum energy intake, with the plant-based meal having the highest satiating effect [34]. Here, there was a large difference in the content of dietary fiber, ranging from 6 g/100 g in the Meat meal to 25 g/100 g in the plant-based meal. This indicates a relationship between the dietary fiber content and the satiating effect of protein, and underlines the complexity of the satiety cascade in meals combining protein and dietary fiber. The mechanisms for this are unknown, although Kehlet et al. [36] showed that the concentration of free amino acids in the blood after a meal with meat and pea fiber was higher than after a meal with meat without dietary fiber, and this might explain part of the interaction between the two components.

## 5. Strength and Limitations

This study has several strengths as well as limitations. First, only men with self-reported gender were included in this study. This limits the generalizability of this study but was chosen to give a more homogenous group of participants, not being influenced by factors such as the menstruation cycle. However, in future studies, a broader range of genders could be included to further generalize the results and to investigate possible gender effects.

Another aspect is the relatively small sample size being 25 participants. Previous sample size power calculations justify the sample size. Furthermore, this study was designed as a very controlled cross-over study controlling the food intake according to energy demand from the previous evening and with control of both beverage and food intake during the full experimental day. In this way, random variations were minimized as much as possible.

The size of the evening meal the day before was calculated based on estimated energy intake. In contrast, all participants received the same test meal. A similar difference in test meal size according to estimated energy intake might have given a more detailed description of the satiating effect of the meals, and could be considered in future studies. 

The IVPD was made using a standardized, validated methodology. However, this methodology does not consider the rate of digestibility, which might affect satiety. In a future study, this aspect would be interesting to investigate further, to better understand the differences in the satiating effect. 

## 6. Conclusions

In conclusion, a ragú “bolognaise” meal with TVP had a higher satiating effect compared with a similar meal with meat and a tendency towards a higher satiating effect compared with a similar meal with soy beans (Green meal) measured by ad libitum energy intake at t = 150 min after the test meal. No difference in the subjective appetite scores was seen. The IVPD was higher in the Meat meal than in the TVP meal, and the in vitro digestibility of the protein itself is therefore not the main explanation for differences in the satiating effect as hypothesized, considering the limitations of the assay. Instead, the lower satiating effect in the Meat meal compared with the TVP meal might be due to the content of ketogenic amino acids and/or the content and composition of dietary fiber in the two meals. The small tendency towards a difference between the Green meal and the TVP meal might partly be explained by the amino acid composition, since the content of ketogenic amino acids was highest in the TVP meal.

Overall, it can be concluded that the satiating effect of plant protein versus meat protein is complex, and our study has shown that, with a similar protein content and close to similar content of dietary fiber, only small differences in satiety are found for the different protein sources, the main difference being the higher long-term satiating effect of the TVP meal.

## Figures and Tables

**Table 1:** Characteristics of the male participants enrolled in this study. Values in mean ± SD, *n* = 25.

Characteristics	Values
Age, years	29 ± 11.3
BMI, kg/m^2^	24.1 ± 2.29
Physical activity level (PAL)	1.68 ± 0.18
Estimated energy intake standardized evening meal, MJ	3.71 ± 0.70

**Table 2:** Ingredients of test meals.

Test Meal	Recipe
TVP	42.5 g dry TVP (83% pea, 17% oats), 200 g chopped tomatoes, 67.5 g water, 62.5 g onion, 3 g garlic, 37.5 g parsnip, 37.5 g soymilk, 35 g tomato puree, 25 g coconut milk, 25 g coconut milk light, 15 g sunflower oil, 7.5 g gastrique, 3.8 g miso, 1.6 g salt, 1 g oregano, 1 g thyme, 1 g smoked paprika, 0.8 g pepper.
Meat	105 g minced beef (3–6% fat), 200 g chopped tomatoes, 87.5 g parsnip, 75 g onion, 3 g garlic, 35 g tomato puree, 17.5 g sunflower oil, 17.5 g water, 8.75 g wheat bran, 7.5 g gastrique, 1.6 g salt, 1 g oregano, 1 g thyme, 1 g smoked paprika, 0.8 g pepper.
Green	143 g soybeans (boiled), 200 g chopped tomatoes, 50 g parsnip, 50 g leek, 3 g garlic, 37.5 g soymilk, 35 g tomato puree, 18.8 g water, 8.75 g sunflower oil, 3.8 g miso, 7.5 g gastrique, 1.6 g salt, 1 g oregano, 1 g thyme, 1 g smoked paprika, 0.8 g pepper.

**Table 3:** Planned meals, beverages and measurements of this study.

	Prior to Test Days	Test Days								
**Time**	**7** **p.m.**	**8** **a.m.**	**8–11.59** **a.m.**	**12.00** **p.m.**	**12.15** **p.m.**	**12.30** **p.m.**	**1.00** **p.m.**	**1.30** **p.m.**	**2.00** **p.m.**	**2.30** **p.m.**
(time from baseline, min)				(0)	(15)	(30)	(60)	(90)	(120)	(150)
**Procedure**										
Standardized evening meal	x									
Standardized breakfast		x								
Test meal				x						
Ad libitum meal										x
Beverage	x ^1^	x	x	x			x			x
VAS				x	x	x	x	x	x	x

^1^ There were no restrictions on water intake at the evening meal, while water intake after the meal until the next morning was restricted to 0.5 L.

**Table 4:** Macronutrients, energy content and weight in the three test meals (ragú “bolognaise” produced with texturized vegetable protein (pea and oat) (TVP), beef meat (Meat) or soy beans (Green). Different letters between samples show a significant difference (*p* < 0.05).

	TVP	Meat	Green	*p* (Difference)
Protein ^1^, % ^2^	6.75	6.30	6.45	0.42
Sum of amino acids ^3^, %	5.59	5.84	5.51	
Fat, %	5.40	5.00	4.65	0.18
Carbohydrate, %	8.8	10.2	8.9	0.17
Dietary fiber, %	4.55 ^ab^	3.75 ^b^	5.55 ^a^	0.02
Water, %	72.9	73.4	73.0	0.50
Energy, kJ/100 g	500	494	478	0.23
Meal weight, g	638	638	642	
Energy/meal, kJ	3190	3152	3069	

^1^ Protein analyzed as N × 6.25. ^2^ % of total fresh weight (g/100 g). ^3^ Sum of protein amino acids from the analysis of total amino acids.

**Table 5:** In vitro protein digestibility (IVPD) and amino acid composition (mg/g protein) of the three test meals (ragú “bolognaise”, produced with texturized vegetable protein (pea and oat) (TVP), beef meat (Meat) or soy beans (Green)).

	WHO ^3^	TVP	Meat	Green
% IVPD, meal		21.05 ^ab^ (1.62 ^4^)	23.49 ^a^ (0.85)	20.17 ^b^ (0.44)
% IVPD, “bolognaise”		20.71 ^b^ (1.02)	30.72 ^a^ (0.21)	22.23 ^b^ (2.18)
Tyr ^1^		35.2	30.4	31.1
Thr ^1,2^	22.7	49.3	38.9	34.7
Leu ^1,2^	59.1	73.9	72.6	69.5
Ile ^1,2^	30.3	42.3	42.2	40.2
Phe ^1^		47.5	40.5	47.5
Trp ^1,2^	6.1	8.8	8.4	11.0
Lys ^1,2^	45.5	59.9	69.3	49.4
Val ^2^	39.4	45.8	43.9	43.9
His ^2^	15.2	22.9	30.4	23.8
Met		10.6	20.3	12.8
Cys		14.1	11.8	16.5
Met + Cys ^2^	22.7	24.6	32.1	29.3
Phe + Tyr ^2^	37.9	82.7	70.9	78.6
Asp		110.9	94.6	106
Ser		37.0	40.5	49.4
Glu		223.6	207.8	255.9
Pro		51.1	50.7	62.2
Gly		40.5	52.4	38.4
Ala		42.3	55.7	40.2
Arg		75.7	69.3	60.3
HPro		0	11.8	0
GABA		8.8	8.4	7.3
Sum of ketogenic amino acids		316.9	302.4	283.4

^1^ Ketogenic amino acids. ^2^ Indispensable amino acids. ^3^ The WHO requirement is given for the indispensable amino acids [26]. ^4^ The average (SD) is given for the IVPD. Different letters within the same row indicate statistical differences (*p* < 0.05).

**Table 6:** Area under/over the curve for hunger, satiety, fullness and prospective eating.

AUC/AOC	TVP	Meat	Green	*p* _(AUC/AOC)_	*p* _(repeated)_
Hunger	9578	9475	9597	0.93	0.90
Satiety	8750	8593	8775	0.87	0.75
Fullness	8669	8335	8727	0.52	0.31
Prospective eating	9149	9034	9186	0.89	0.95

**Table 7:** Ad libitum energy intake at t = 150 min. Different letters between samples show a significant difference (*p* < 0.05).

	TVP	Meat	Green	*p* _(ad lib)_	Std. Err
Ad libitum intake, kJ	758 ^b^	957 ^a^	903 ^ab^	0.02	95.4

## Data Availability

All data and codes are available by request.

## Supplementary Materials

The following supporting information can be downloaded at https://www.mdpi.com/article/10.3390/nu16193407/s1: Table S1: Description of subjective appetite questions given to the participants; Table S2:Concentration of free α-amino groups per gram of protein in samples before in vitro digestion, during the pepsin and pancreatin digestion step. Results for both pepsin and pancreatin steps were corrected for values before digestion.
