# Supplementary material for: The Satiating Effect of Extruded Plant Protein Compared with Native Plant and Meat Protein in a Ragú “Bolognaise” Meal: A Randomized Cross-Over Study
Source: Nutrients. 2024 Oct 8;16(19):3407. doi: 10.3390/nu16193407 (PMC11479086; doi:10.3390/nu16193407)
Supplement: Supplementary file 1 [file nutrients-16-03407-s001.zip › nutrients-3213645-supplementary.pdf]

Supplementary Materials Table S1

Description of subjective appetite questions given to the participants	
Satiety	Satiety is the feeling you experience throughout the entire body after eating – in other words, when your energy reserves have been replenished.
Hunger	Hunger is a feeling that combines the sensation of energy in the body with a feeling of emptiness in the stomach.
Fullness	Fullness is centred around how much you perceive the stomach to be filled. It is more of a physical sensation than a feeling of energy.
Prospective food intake	How much you want to eat is a feeling which is centred around how you think about food and not about a sensation of energy in the body.

Supplementary Materials Table S2

Concentration of free α-amino groups per gram of protein in samples before in vitro digestion, during the pepsin and pancreatin digestion step. **Free α-amino groups (μM/g protein)**

**Sample**	**Before digestion**		**Pepsin 2h corrected**		**Pancreatin 2h corrected**	
	Mean	SD	Mean	SD	Mean	SD
TVP meal	5.37^{b}	0.47	2.64^{bc}	0.70	13.05^{b}	1.70
TVP bolognaise	5.22^{b}	0.77	3.49^{ab}	0.79	12.00^{b}	1.04
Meat meal	4.43^{b}	0.66	4.72^{a}	0.70	14.35^{b}	0.89
Meat bolognaise	7.74^{a}	0.32	4.16^{ab}	0.99	18.81^{a}	0.96
Green meal	4.37^{b}	0.23	3.38^{ab}	0.42	12.42^{b}	0.56
Green bolognaise	5.14^{b}	0.11	1.48^{c}	0.73	15.61^{ab}	2.29
Bovin Serum Albumin (BSA)	0.57	0.09	6.97	0.37	19.48	2.09

Results are shown as mean and standard deviation (n = 3). Different letters in the same column represent significant differences. One-Way ANOVA followed by post hoc Tukey test ($p<0.05$). Results obtained after the 2h in the gastric phase and after 4h digestion (gastric + intestinal phase)

have been corrected for the amount of the free amino groups detected before digestion. The intestinal phase was calculated by subtracting the total digestibility (4h) from the digestibility in the gastric phase. Bovin serum albumin was used as an internal standard representing a highly digestible protein.
